# Use of My Health Record by Clinicians in the Emergency Department: An Analysis of Log Data

**DOI:** 10.3389/fdgth.2021.725300

**Published:** 2021-08-20

**Authors:** Alexandra K. Mullins, Heather Morris, Joanne Enticott, Michael Ben-Meir, David Rankin, Kumar Mantripragada, Helen Skouteris

**Affiliations:** ^1^Health and Social Care Unit, School of Public Health and Preventative Medicine, Monash University, Melbourne, VIC, Australia; ^2^Austin Health, Melbourne, VIC, Australia; ^3^Cabrini Health, Melbourne, VIC, Australia; ^4^Warwick Business School, University of Warwick, Coventry, United Kingdom

**Keywords:** electronic health record, health information exchange, emergency department, observational study, patient admission

## Abstract

**Objectives:** Leverage log data to explore access to My Health Record (MHR), the national electronic health record of Australia, by clinicians in the emergency department.

**Materials and Methods:** A retrospective analysis was conducted using secondary routinely-collected data. Log data pertaining to all patients who presented to the emergency department between 2019 and 2021 of a not-for-profit hospital (that annually observes 23,000 emergency department presentations) were included in this research. Attendance data and human resources data were linked with MHR log data. The primary outcome was a dichotomous variable that indicated whether the MHR of a patient was accessed. Logistic regression facilitated the exploration of factors (user role, day of the week, and month) associated with access.

**Results:** My Health Record was accessed by a pharmacist, doctor, or nurse in 19.60% (*n* = 9,262) of all emergency department presentations. Access was dominated by pharmacists (18.31%, *n* = 8,656). All users demonstrated a small, yet significant, increase in access every month (odds ratio = 1.07, 95% Confidence interval: 1.06–1.07, *p* ≤ 0.001).

**Discussion:** Doctors, pharmacists, and nurses are increasingly accessing MHR. Based on this research, substantially more pharmacists appear to be accessing MHR, compared to other user groups. However, only one in every five patients who present to the emergency department have their MHR accessed, thereby indicating a need to accelerate and encourage the adoption and access of MHR by clinicians.

## Introduction

An electronic health record (EHR) is defined as a longitudinal digital record of patient health information that patients and authorized healthcare professionals can access, manage, and upload health information to (1). EHRs have been adopted by most of the upper-middle and high-income countries worldwide, including Australia (2), in anticipation of their potential to improve efficiency, patient care, and safety in the field of medicine (3). The national personally controlled EHR of Australia, known as My Health Record (MHR), was introduced in Australia as an opt-in system in 2012 and transitioned to an opt-out scheme in 2019 (4). As of the opt-out date, in January 2019, 90% of all Australians had an MHR (MHR can include patient information such as allergies, medicines, pathology reports, diagnostic scans, and discharge summaries) (4).

The emergency department (ED) has been a major focus for the effective implementation and use of EHRs (5), as ED clinicians^1^ require efficient access to patient information that may exist outside of what is available within the internal medical record system of a healthcare provider (7). The benefits of using an EHR in the ED include improved communication to prevent medication errors, increased efficiencies (8), and improved coordination among healthcare providers (9). Despite the benefits associated with the use of EHRs in the ED, their usage is low (5). Surveys and interviews conducted within a private hospital in Melbourne, Australia, suggest that MHR had been accessed at least once by 50% of the clinicians (8). There is no record of an objective analysis of MHR access having been conducted in Australia, though it is a critical requirement in order to understand who uses MHR and how it is used.

Understanding the predictors of MHR access and use is crucial to improve usage (10). Previous research, exploring EHR systems that exist outside of Australia, highlights that the key predictors of EHR access in the ED include: repeat patient visits; patients with comorbidities and patients with known data in the exchange (11). Authors Johnson and Unertl (11) also highlighted that the healthcare sites with nurses, clerks, and physicians accessing the system have the highest levels of access. There has been minimal research on the impact of the type of user, the time of day, or the day of the week on the use of MHR in the ED, though the abovementioned factors are all important considering that more than 40% of the ED presentations in Australia occur outside of normal business hours^2^ (when access to information about a patient from external healthcare services may be unavailable and when the number and type of staff available in the ED may differ from normal hours) (13).

Real-time user log data has been used in this study from the perspective of user-initiated sessions to provide an objective insight into MHR access. This study aims to explore the association of MHR access during patient care in the ED with user type, time of day, and day of the week.

## Materials and Methods

This study is reported in accordance with the Reporting of studies Conducted using Observational Routinely-collected health Data (RECORD) Statement (14).

### Study Design and Setting

A retrospective cohort design was employed including secondary analysis of routinely-collected log data from January 1, 2019 to December 31, 2020. The data were routinely collected by Cabrini Health, a not-for-profit, private acute health service in Melbourne, Australia. Cabrini Health treats more than 88,000 patients each year (including 23,000 ED presentations) and comprises a large acute teaching hospital with an ED, a second smaller acute hospital, an aged care facility, a palliative care facility, and community-based services.

### Sample Frame

The study sample drawn from the administrative database of Cabrini Health included all individuals who presented to the ED between January 2019 and December 2020.

### Outcome Measures

The primary outcome (dependent variable) in this study was measured using a binary variable (1= MHR access and 0 = MHR not accessed) by a clinician (pharmacist, doctor, or nurse), during the time period when the patient presented to, and was discharged from, the ED. For the purpose of this research, the term MHR access has been used to describe the act of logging into the MHR of a patient with the intention of retrieving information.

Based on a review of the literature, two independent variables were considered in this study, which include the month (as a continuous variable) and the day of the week (as a categorical variable). The month was included to account for the changes over time in MHR access, while the day of the week was included to account for the impact of the ED presentations occurring outside of normal business hours (weekends) when general practices and other health services are usually closed (13). Three different groups of clinicians (pharmacists, doctors and nurses) were included and compared in this research to explore differences in access across groups and to gain a deeper understanding of MHR access where little is known about how MHR is accessed and used to date (i.e. nurses) (8).

### My Health Record

My Health Record comprises several web pages, including a health record overview, information on medicines, event and discharge summaries, pathology reports, diagnostic imaging requests, letters from specialists, referrals, and shared health summaries^3^. MHR encompasses information uploaded by an array of healthcare providers across healthcare systems, therefore the data within differs from what is available on the internal medical record at Cabrini^4^, or any other health service. For example, the prescription and dispense view within a patient's MHR can include information such as the name of the pharmacy that the patient frequently visits and the medications dispensed by that pharmacy, which may otherwise be unavailable on the internal electronic medical record at the hospital.

### Data Sources and Linkage

**Figure 1:**
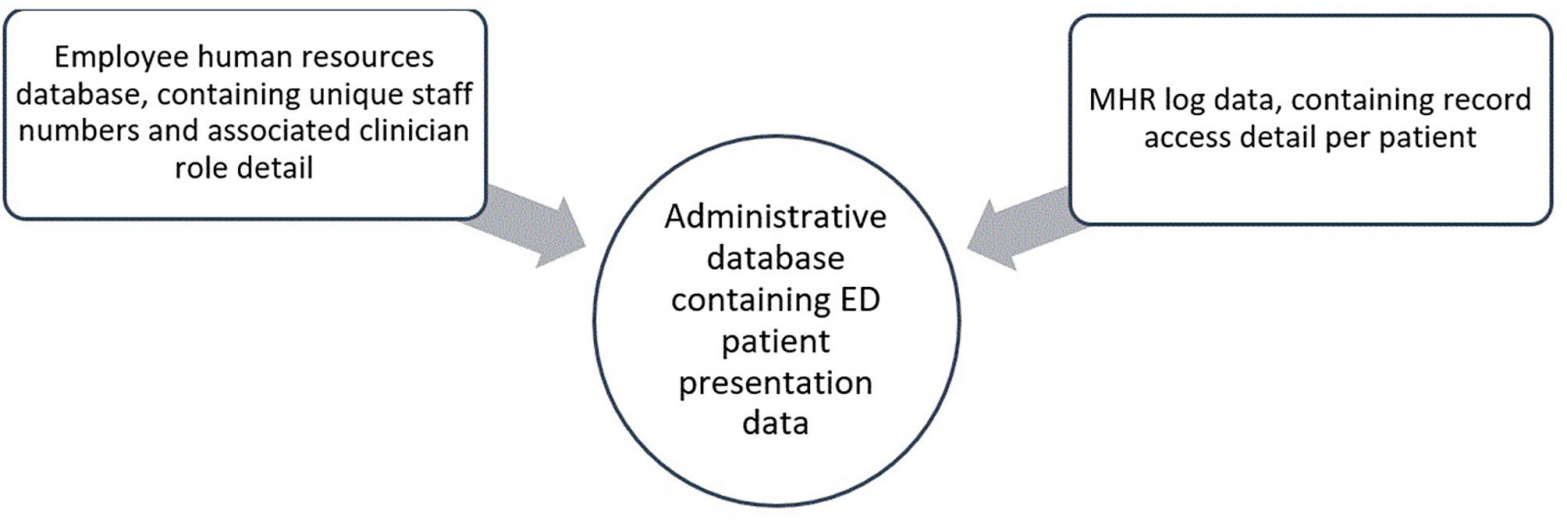
Visual representation of the three databases that were linked in this study.

The administrative data were linked to two separate databases at the patient level, including MHR log data and employee human resources data (see Figure 1 for a graphical representation of the linked data at each stage).

#### The Administrative Database

The administrative database contained the patient ID, the admission date and time, and the discharge date and time of every patient who presented to the ED.

#### MHR Log Data

**Figure 2:**
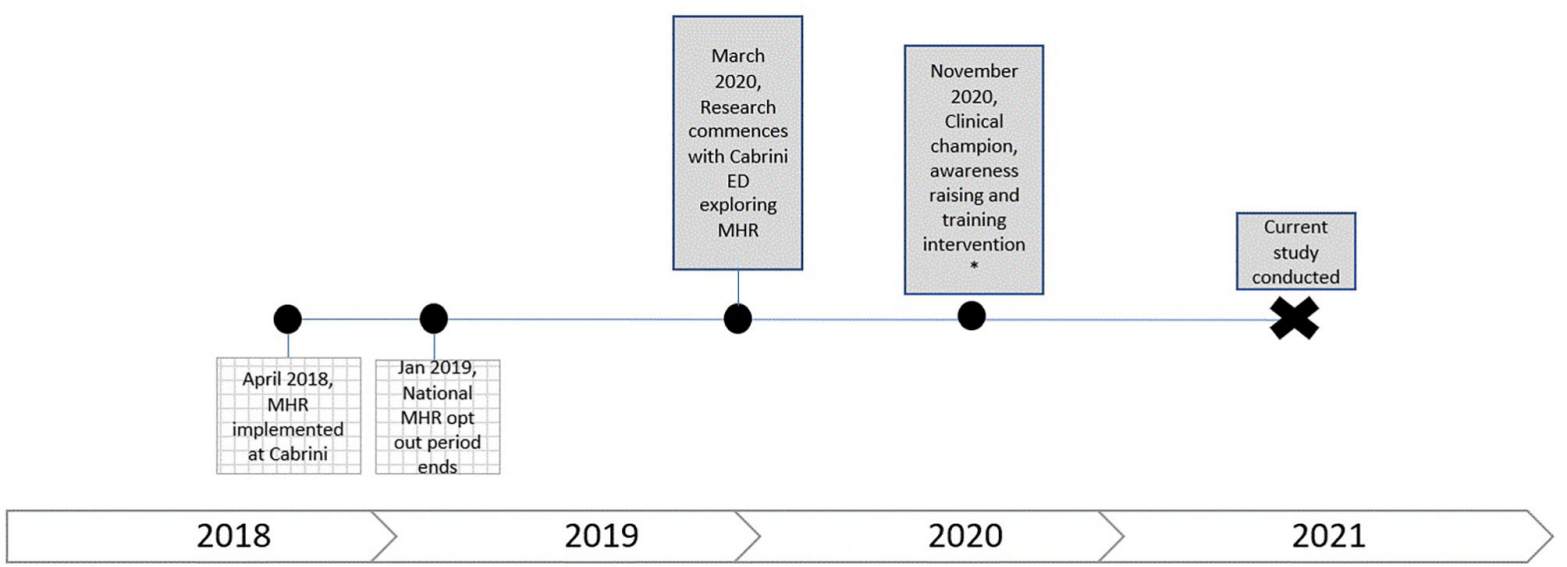
Visual representation of MHR implementation at Cabrini relative to the time period when this study was conducted.

My Health Record, made available through the Cabrini patient administration system in April 2018 (see Figure 2 for visual representation), is accessed at Cabrini through a password-protected icon (supported by a one-click direct access from the hospital-based patient file, only when a patient record is available). As mentioned earlier, MHR comprises several web pages. Action by one user (for example, clicking a button to download the pharmacy information related to a patient) leads to multiple server requests that are documented in the log data file.

The original log dataset included a page uniform resource locator (URL) representing MHR access, patient ID, user ID, day, and time-stamp. To ensure that the data within the log files were representative of the user's interaction with MHR, a series of tests were completed (that ensured the URLs generated corresponded to MHR access by the correct user).

#### Employee Human Resources Data

Employee human resources data included the role and level of experience of each clinician at the ED (linked *via* a unique clinician user ID).

### Statistical Analysis

Descriptive analysis was used to determine the frequency of ED presentations and the prevalence of MHR access.

Logistic regression was used to explore the primary outcome (0, 1) by time (month 1–24, continuous variable) and day of the week (1–7, categorical variable). This was done for all user groups (pharmacists, doctors, and nurses), and was repeated for each user group separately to investigate the differences in MHR access by the user groups. Effect size estimates, representing the magnitude of differences in MHR usage within subgroups, are presented as odds ratios (OR) with 95% confidence intervals (CIs). Logistic regression was the method of choice as it is a common statistical technique used for analyzing binary outcomes (15). Comparisons were made (independent *t*-tests) among the three user groups for relevant outcomes.

Descriptive statistics were analyzed using the Statistical Package for the Social Sciences (SPSS TM version 19.0; Chicago, IL, USA) and regression with Stata V16. Statistical significance was set as *p* < 0.05.

### Ethics

The protocol for the study was reviewed and approved by the Cabrini Research ethics committee at the study site in January 2021 (reference number 01-20-04-20). The study was carried out in compliance with the Declaration of Helsinki.

## Results

### Frequency of MHR Access per Patient Admission

A total of 47,266 patients presented to the ED between January 1, 2019 and December 31, 2020. The MHR was viewed by at least one clinician (pharmacist, doctor, or nurse) in 19.60% (*n* = 9,262) of all the admissions to the ED, of which 18.31% (*n* = 8,656), 2.88% (*n* = 1,360), and 0.47% (*n* = 220) of the patients who presented had their MHR accessed by a pharmacist and/or a doctor and/or a nurse, respectively. A total of only 0.07% of the patients (*n* = 34) had their MHR accessed by all clinician types (pharmacist, doctor, and nurse), refer to Table 1.

**Table 1:** Descriptive data for MHR access by each clinician.

	**Clinician MHR accessed by**				
	**Pharmacist**	**Doctor**	**Nurse**	***n***	**%**
	Y	Y	Y	34	0.07%
	Y	Y	N	777	1.64%
	Y	N	Y	121	0.26%
	Y	N	N	7,724	16.34%
	N	N	Y	57	0.12%
	N	N	N	38,004	80.40%
	N	Y	Y	8	0.02%
	N	Y	N	541	1.14%
Total^*^				9,262	19.60%
All^+^				47,266	100%

*Y = the patient had his/her MHR accessed by one of a pharmacist, nurse, or doctor (as indicated). N = the patient did not have his/her MHR accessed by one of a pharmacist, nurse, or doctor (as indicated)*.

* *Total of all patients who presented to the ED and had their MHR accessed by at least one clinician*.

+ *All patients who presented to the ED during the study period*.

### Predictors of Increased Use of MHR

Logistic regression revealed that the explanatory variables, month and day of the week, were significantly associated with MHR access per patient admission to the ED (see Table 2). Increase in time by one month, increased the odds of MHR access by 7% (odds ratio [OR] = 1.07, 95% CI: 1.06–1.07, *p* ≤ 0.001). MHR access was more likely to occur between Monday and Friday compared to Sunday (see Table 2). There was no significant difference between MHR access on Saturday and Sunday (OR = 0.88, 95% CI: 0.79–0.96). Supplementary Table 1 provides descriptive data.

**Table 2:** Outputs for the four regression models using data from patients (*n* = 47,266) who presented to the ED between January 1, 2019 and December 31, 2020.

	**MHR accessed by**							
	***pharmacist, doctor or nurse***		***pharmacist***		***doctor***		***nurse***	
**Explanatory variable**	**OR (95% CI)**	***P* value**	**OR (95% CI)**	***P* value**	**OR (95% CI)**	***P* value**	**OR (95% CI)**	***P* value**
Day of the week								
Sunday (R)	1	**–**	1	**–**	1	**–**	1	**–**
Monday	1.26(1.15–1.38)	<0.001^*^	1.31(1.19–1.43)	<0.001^*^	0.84(0.69–1.03)	0.001	1.01(0.58–1.78)	0.967
Tuesday	1.25(1.14–1.36)	<0.001^*^	1.29(1.18–1.42)	<0.001^*^	0.79(0.64–0.97)	0.009	0.96(0.54–1.72)	0.894
Wednesday	1.56(1.43–1.71)	<0.001^*^	1.58(1.45–1.74)	<0.001^*^	1.08(0.89–1.31)	0.099	1.47(0.87–2.51)	0.152
Thursday	1.67(1.53–1.82)	<0.001^*^	1.70(1.55–1.86)	<0.001^*^	1.24(1.03–1.49)	0.557	2.32(1.43–3.78)	0.001
Friday	1.32(1.20–1.44)	<0.001^*^	1.39(1.26–1.52)	<0.001^*^	0.80(0.65–0.98)	0.026	1.95(1.18–3.21)	0.009
Saturday	0.88 (0.79–0.96)	0.006	0.93(0.84–1.02)	0.126	0.61(0.49–0.77)	<0.001^*^	0.51(0.26–1.03)	0.061
Time (Month)	1.07 (1.06–1.07)	<0.001^*^	1.06 (1.06–1.07)	<0.001^*^	1.07 (1.06–1.08)	<0.001^*^	1.05 (1.03–1.07)	<0.001^*^

*OR = odds ratio; CI = confidence interval*;

* *significant at p < 0.001; (R) = Reference*.

Both month and day of the week (except Saturday) were significantly associated with the use of MHR by pharmacists. Increase in time by 1 month, increases the odds of pharmacists using MHR by 6% (OR = 1.06, 95% CI: 1.06–1.07, *p* < 0.001). Pharmacists were significantly more likely to use MHR between Monday and Friday compared to Sunday (see Table 2). There was no significant difference between MHR access by pharmacists on Saturday and Sunday (OR= 0.93, 95% CI: 0.84–1.02).

Both month and day of the week (Only Saturday) were significantly associated with the use of MHR by doctors in the ED (presented in Table 2). Increase in time by one month, increases the odds of doctors using MHR by 7% (OR = 1.07, 95% CI: 1.06–1.08, *p* < 0.001). Doctors were significantly less likely to use MHR on a Saturday than on a Sunday (OR = 0.61, 95% CI: 0.49–0.77, *p* < 0.001). There was no significant difference in MHR access by doctors between Monday and Friday when compared to Sunday.

Only the explanatory variable, month, was significantly associated with the use of MHR by nurses in the ED (presented in Table 2). Increase in time by one month, increases the odds of nurses using MHR by 5% (OR = 1.05, 95% CI: 1.03–1.07, *p* < 0.001). There was no significant difference in MHR access by nurses between Monday and Saturday when compared to Sunday.

**Figure 3:**
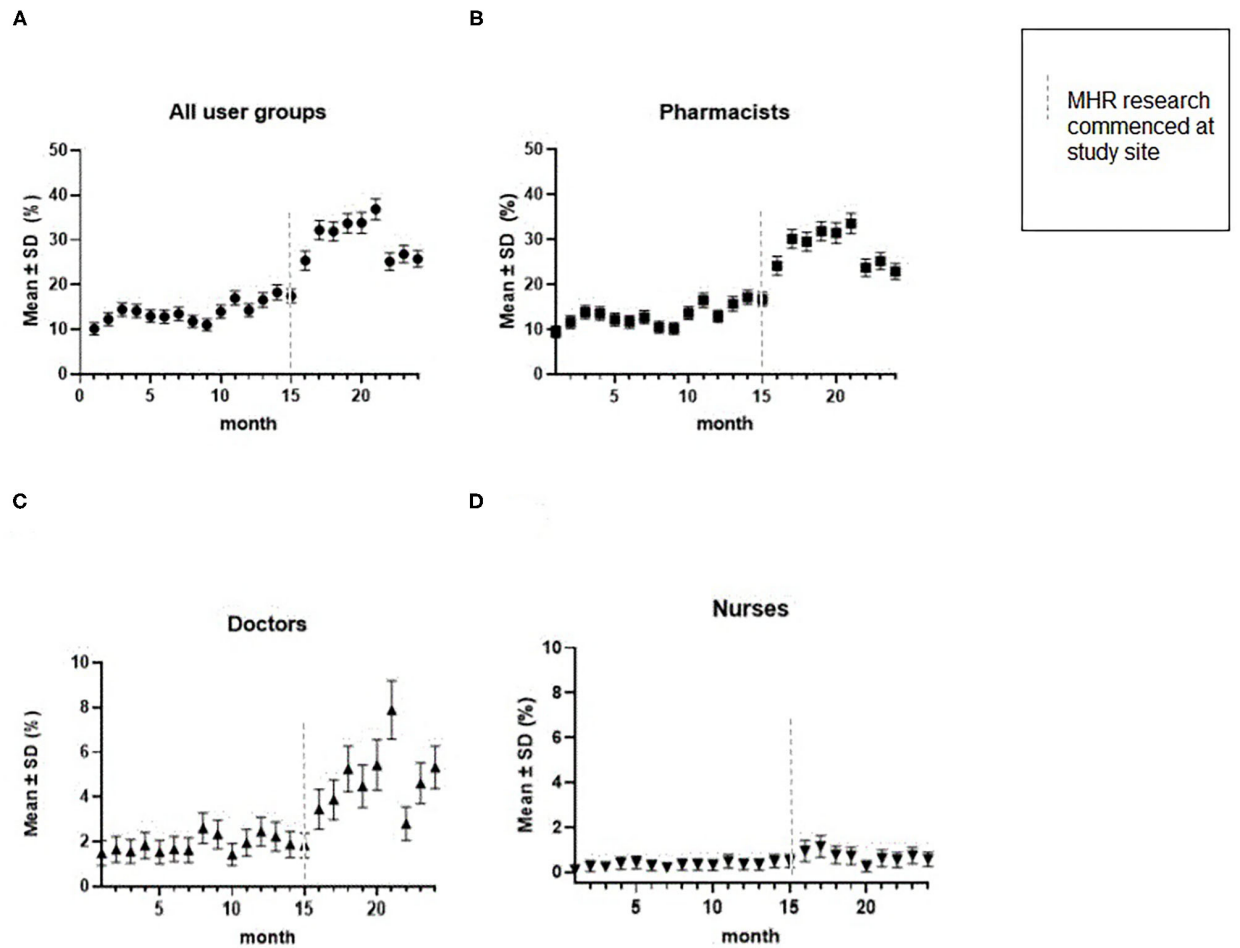
Mean rate of MHR access by month by a clinician, as a proportion of all patients who presented to the ED, broken down by user group. **(A)** is the rate of MHR access by all three user groups (pharmacists, doctors, and nurses); **(B)** is the rate of MHR access by pharmacists; **(C)** is the rate of MHR access by doctors; and **(D)** is the rate of MHR access by nurses. MHR research at the study site involved surveys and interviews with ED pharmacists and physicians to understand MHR use. As a result of the findings (see Mullins, Mousa (16) for further detail), where clinicians flagged further training and awareness of MHR was required, an educational video was produced and circulated and two clinical champions were recruited to address the previously mentioned barriers to use. Growth in use increases progressively from month 15 to month 20, which is likely driven by an increased awareness of the MHR system, driven by the commencement of the MHR quality improvement research at the study site. Specifically, the production of the educational video took place in September 2020, where clinical champions (a pharmacist and a doctor) were actively using and promoting MHR, in preparation for and during filming; this likely explains the peak use of MHR in September 2020 and the drop-off thereafter.

Across the 2-year study period, the regression model indicated an upward trend in MHR access. To highlight this finding, the mean rate of MHR access by month was plotted for each clinical user group (Figure 3). For MHR access involving pharmacists and doctors, a pronounced upward trend in MHR access, from January 2019 to December 2020, was observed. Over the same time period, a slight upward trend was observed for MHR access involving nurses.

**Figure 4:**
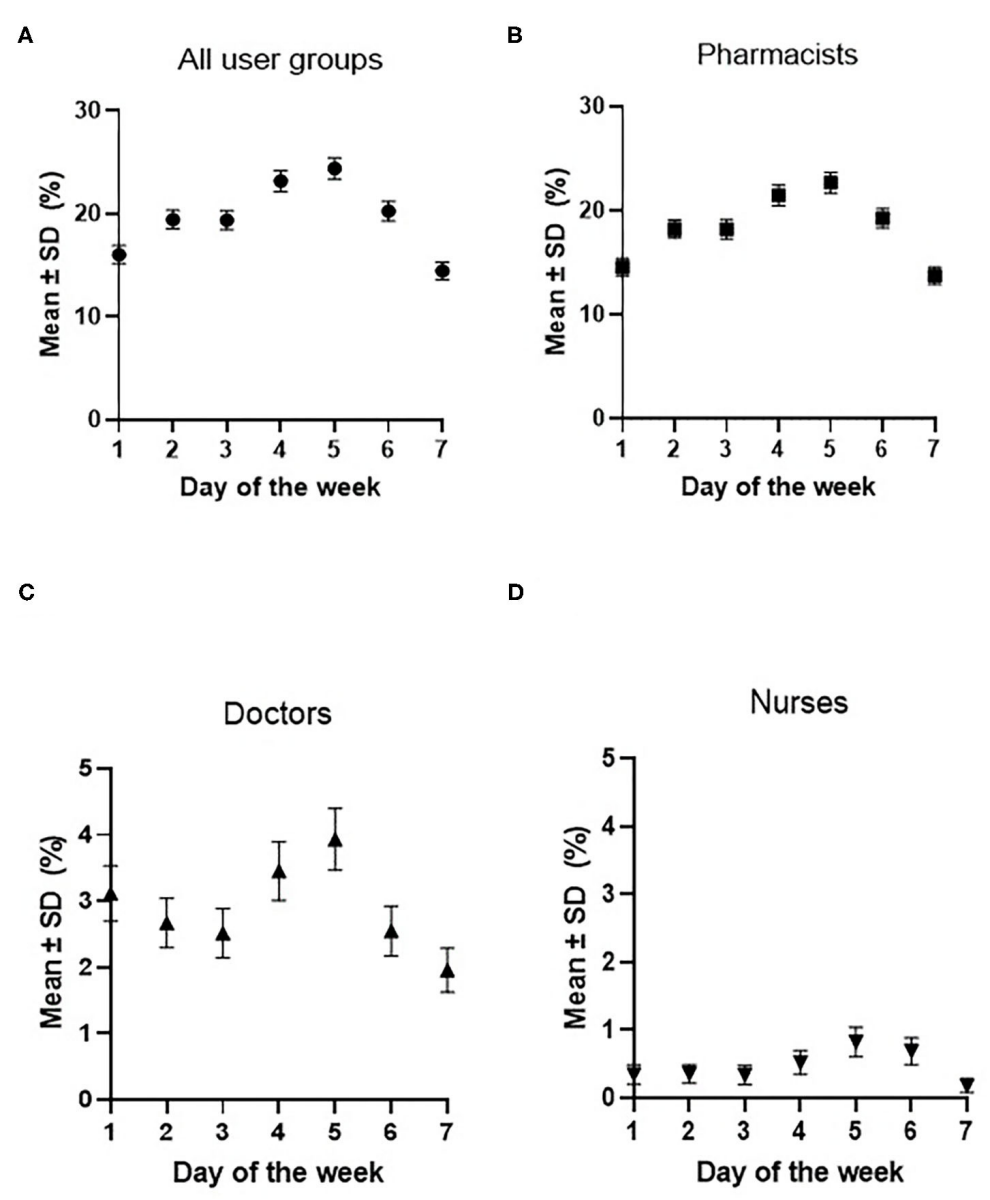
Mean rate of MHR access per day, as a proportion of all patients who presented to the ED, by user group. **(A)** is the rate of MHR access by all three user groups (pharmacists, doctors, and nurses); **(B)** is the rate of MHR access by pharmacists; **(C)** is the rate of MHR access by doctors; and **(D)** is the rate of MHR access by nurses. Error bars show the 95% confidence intervals.

Regression modeling indicated that MHR was more likely to be accessed between Monday and Friday compared to Sunday. To highlight this finding, the mean rate of MHR access per day was plotted for each clinical user group (Figure 4). In instances where pharmacists used MHR, a pronounced upward trend between Monday and Friday was observed, with MHR access decreasing between Saturday and Sunday. For pharmacists, doctors, and nurses, the highest proportion of access of MHR occurred on Thursdays and the least was on Saturdays.

## Discussion

According to our knowledge, this study is the first to leverage log data, linked to routinely collected ED data, which explores MHR access by clinicians in an Australian ED. We found that the proportion of all patients who presented to the ED and had their MHR accessed by a clinician is strikingly low (19.60%), occurring mostly during the weekdays than on the weekends. Albeit, the access rates reported here are consistent with those reported previously regarding EHRs in Israel and the United States (5, 16), emphasizing that individual clinicians do not always use EHR systems once their healthcare service has adopted them (17). A possible explanation is that there may be no perceived need to view the medical history of the patient, for example, when a patient presents with a problem of mild severity, like a broken finger (18).

Despite the fact that access rates of MHR are low, our analyses show that the overall MHR access in the ED has tripled over the course of this study, increasing significantly each month. One possible explanation is that the perception of the usefulness and the ease-of-use of MHR is increasing, subsequently leading to an increase in the access of MHR (19).

The use of MHR by ED clinicians in this study was more likely to occur between Monday and Friday, which was anticipated as there is a reduced number of staff on weekends vs. weekdays at the study site (20). Across all clinicians, the highest use of MHR occurred on Thursdays. This may be explained by the fact that ED admissions on an average in Australia (21), and in this study (when compared to other weekdays only), occurred the least on Wednesdays and Thursdays, suggesting that clinicians may use MHR more frequently when they have the time to do so. Recent research by the authors Vehko and Hyppönen (20) suggests that EHRs can lead to increased levels of psychological distress and burnout (21) among clinicians when they are perceived as unreliable or not user-friendly. Therefore, clinicians in our study may have avoided using MHR at busy times because it compounds their anxiety and stress. By supporting clinicians to gain confidence in EHR use [through necessary training and clinical application (22)], healthcare providers are more likely to gain buy-in from clinicians, avoid the risk of distress and see EHR use improve (23) - as clinicians see benefit in leveraging the platform to facilitate their practice, especially in stressful environments.

Overall, MHR access in this research was dominated by pharmacists, in accordance with the authors Mullins and Mousa (8) who reported that pharmacists are the most frequent users of MHR. These results add further weight to the argument that pharmacists are early information system adopters (24). Early adoption may be explained by the role pharmacists play in the management of a patient's medication (including preventing medical errors and the subsequent risks to patients) and the opportunity that MHR presents pharmacists with to fulfill this role by supporting access to a patient's medical history (25). Further research is required to explore how the quantity and quality of information within MHR impacts the decision of clinicians to access or avoid the MHR system.

The current study had several limitations. We could not control for the hours worked by each clinician in the ED. Therefore, the use of MHR by pharmacists, compared to the nurses and doctors, may be even more pronounced when hours worked are controlled for (given less pharmacists work in the ED at the study site each day, than doctors or nurses) (26). As a result, MHR access rates favored weekdays for pharmacists when the number of staff working was high. Secondly, this study only explored a clinician's role based on the day of the week and month. Exploring other predictors of use (for example, triage category and/or condition complexity) and a clinician's motivation to use MHR (for example, to improve diagnosis accuracy and/or to avoid test duplication) may enhance patient care and improve efficiencies for patients, clinicians, and the healthcare system in a more broader sense (27). Finally, accessing the MHR system does not mean that the data viewed impacted patient care. This may be a limitation associated with the use of secondary data, yet it does not impact the results (28).

Further research is required to explore the use of MHR outside of the health service included in this study. This is particularly pertinent since the use of MHR may be dependent on the type or size of the healthcare organization (29). Moreover, future research that explores what motivates different healthcare groups, including clinicians, healthcare providers and patients, to use MHR is required (such as patient care improvements, administration process improvements, and patient interest in managing their health and care) and may accelerate MHR uptake across user groups.

Overall, this study highlighted a small, yet significant, increase in MHR access by pharmacists, doctors, and nurses between January 2019 and December 2020. While pharmacists were responsible for the majority of MHR access, it has been observed that MHR was accessed for only one in every five patients who presented to the ED. This research highlights a desperate need to accelerate and encourage MHR adoption and access by all clinicians in the ED. Given this research included only one site, further research across other EDs is required for generalized conclusions to be drawn.

## Data Availability Statement

The raw data supporting the conclusions of this article will be made available by the authors, without undue reservation.

## Author Contributions

All authors made contributions to all of the following: (1) the conception and design of the study, acquisition of data, and analysis and interpretation of data, (2) drafting the article and revising it critically for important intellectual content, and (3) approval of the version to be submitted.

## Conflict of Interest

The authors declare that the research was conducted in the absence of any commercial or financial relationships that could be construed as a potential conflict of interest.

## Publisher's Note

All claims expressed in this article are solely those of the authors and do not necessarily represent those of their affiliated organizations, or those of the publisher, the editors and the reviewers. Any product that may be evaluated in this article, or claim that may be made by its manufacturer, is not guaranteed or endorsed by the publisher.

## Footnotes

^1^The term clinician “refers to a healthcare professional qualified in the clinical practice of medicine” (6). CMS. Quality Measures & You: Clinicians 2019 [Available from: https://www.cms.gov/Medicare/Quality-Initiatives-Patient-Assessment-Instruments/MMS/QMY-Clinicians].

^2^In-hours include: weekdays from 8:00 a.m. to 7:59 p.m. (excluding public holidays) and Saturdays from 8:00 a.m. to 12:59 p.m. (excluding public holidays). After-hours include: weekdays before 8:00 a.m. and after 7:59 p.m., Saturdays before 8:00 a.m. and after 12:59 p.m., and all Sundays and public holidays (12). AIHW. Use of emergency departments for lower urgency care: 2015–16 to 2018–19 Technical Note. 2020.

^3^Under the MHR Act, healthcare providers are authorized to upload information to the MHR system. Cabrini automatically uploads discharge summaries to the patient's MHR.

^4^Cabrini clinicians typically use the Electronic Web-based Patient Administration System to access information about a patient (which is only available when a patient has been admitted previously). Information within the Patient Administration System typically includes demographic information about a patient, details about the procedure, medications prescribed to the patient, and tests performed (this information and more is also available in the paper record file, stored offsite). From April 1, 2020, ED pharmacists are also required to check SafeScript (electronic software that allows prescribing and dispensing records for high-risk medicines to be transmitted in real-time to a centralised database). when the monitored medicine supplied is intended to be taken by the patient outside of the hospital. Clinicians also have access to patient reports and images via I-MED Online and pathology results *via* a web-based application, Sonix Dx.
