# Supplementary material for: Use of My Health Record by Clinicians in the Emergency Department: An Analysis of Log Data
Source: Front Digit Health. 2021 Aug 20;3:725300. doi: 10.3389/fdgth.2021.725300 (PMC8521888; doi:10.3389/fdgth.2021.725300)
Supplement: Supplementary file 1 [file Table_1.docx]

Supplementary Table 1. Numbers and mean proportions (SD, standard deviations) for MHR use per patient presentation by day of the week. Note: some ED presentations had a patient MHR accessed by more than one clinician (see Table 1).

|  | MHR accessed by | | | | | | | | | | | | | |
| --- | --- | --- | --- | --- | --- | --- | --- | --- | --- | --- | --- | --- | --- | --- |
|  | | **Total**  **ED presentations** | ***pharmacist, doctor or nurse*** | | | ***pharmacist*** | | | ***doctor*** | | | ***nurse*** | | |
|  |  |  | n | mean  proportion | SD | n | mean  proportion | SD | n | mean  proportion | SD | n | mean  proportion | SD |
| Day of the week | |  |  |  |  |  |  |  |  |  |  |  |  |  |
| Sunday (R) | | 6,581 | 1,055 | 0.160 | 0.367 | 958 | 0.146 | 0.353 | 205 | 0.031 | 0.174 | 23 | 0.003 | 0.059 |
| Monday | | 7,305 | 1,420 | 0.194 | 0.396 | 1,333 | 0.182 | 0.386 | 195 | 0.027 | 0.161 | 26 | 0.004 | 0.060 |
| Tuesday | | 6,755 | 1,310 | 0.194 | 0.395 | 1,230 | 0.182 | 0.386 | 170 | 0.025 | 0.157 | 23 | 0.003 | 0.058 |
| Wednesday | | 6,487 | 1,504 | 0.232 | 0.422 | 1,393 | 0.215 | 0.411 | 224 | 0.035 | 0.183 | 34 | 0.005 | 0.072 |
| Thursday | | 6,666 | 1,628 | 0.244 | 0.430 | 1,513 | 0.227 | 0.419 | 262 | 0.039 | 0.194 | 55 | 0.008 | 0.090 |
| Friday | | 6,829 | 1,384 | 0.203 | 0.402 | 1,316 | 0.193 | 0.394 | 174 | 0.025 | 0.158 | 47 | 0.007 | 0.083 |
| Saturday | | 6,643 | 961 | 0.145 | 0.352 | 913 | 0.137 | 0.344 | 130 | 0.020 | 0.139 | 12 | 0.002 | 0.042 |
| Total | | 47,266 | 9,262 | 0.196 | 0.397 | 8,656 | 0.183 | 0.387 | 1,360 | 0.029 | 0.167 | 220 | 0.005 | 0.068 |
